# Climates on incidence of childhood type 1 diabetes mellitus in 72 countries

**DOI:** 10.1038/s41598-017-12954-8

**Published:** 2017-10-09

**Authors:** Yin-ling Chen, Yong-cheng Huang, Yong-chao Qiao, Wei Ling, Yan-hong Pan, Li-jun Geng, Jian-long Xiao, Xiao-xi Zhang, Hai-lu Zhao

**Affiliations:** 1 Center of Diabetic Systems Medicine, Guangxi Key Laboratory of Excellence, Guilin Medical University, Guilin, 541004 China; 2 Department of Immunology, Xiangya School of Medicine, Central South University, Changsha, Hunan 410078 China; 3 Department of Immunology, Faculty of basic Medicine, Guilin Medical University, Guilin, 541004 China

## Abstract

We are aimed to systematically assess the worldwide trend in incidence of childhood type 1 diabetes mellitus (CT1DM) from 1965 to 2012 and to discuss whether climate affect incidence of CT1DM. We searched the relevant literatures in detail to judge the effect of different climates on incidence of CT1DM. The climates included Mediterranean, monsoon, oceanic, continental, savanna, and rainforest. According to different climates, we further researched relevant factor such as sunshine durations and latitudes. The overall incidence of CT1DM in 72 countries was 11.43 (95% CI 10.31–12.55) per 100,000 children/yr. The incidence of CT1DM in Oceanic climate [10.56 (8.69–12.42)] is highest compared with other climates; the incidence in 40°–66°34′N/S [14.71 (12.30–17.29)] is higher than other latitude groups; the incidence in sunshine durations with 3–4 hours per day [15.17 (11.14–19.20)] is highest compared with other two groups; the incidence of CT1DM from 2000 to 2012 [19.58 (14.55–24.60)] is higher than other periods; all *p* < 0.01. Incidence of CT1DM was increasing from 1965 to 2012, but incidence in Oceanic climate is higher than other climates. Furthermore, it is higher in centers with higher latitude and lower sunshine durations. The climates might play a key role in inducing CT1DM.

## Introduction

The worldwide variation in the incidence of type 1 diabetes mellitus (TIDM) among children has been confirmed to be increased over the past 50 years^1–3^, especially among children of 10–14 years of age^4^. Childhood type 1 diabetes mellitus (CTIDM) is a syndrome caused by β-cell destruction that results in progressive or acute insulin deficiency^5,6^.

While we know that children with diabetes aged less than 7 years are at high risk of cognitive dysfunction, and poor glycaemic control might induce hypoglycaemia that could influence the developing nervous system^7,8^. Furthermore, immunosuppressive drugs for CT1DM treatment have kidney toxicity and other side effects^9^.

No clear evidence of a correlation between the CT1DM and climates had emerged from human or animal studies. Previous studies indicated the milk consumption^10,11^, dietary habit^12,13^, socioeconomic^14^, latitude^15^, familial predisposition^16^, drinking water^17^ or radiation^18^ might be important factor for CT1DM.

It is vital therefore to conduct this study to confirm the various climates in relation to the incidence of CT1DM.

## Methods

### Data collection

This study is supported by the Guilin Medical University Ethnic Committee Board. Articles published between Jan 1, 1965 and Jan 31, 2017 that were systematically searched in the databases: the PubMed, the Chinese National Knowledge Infrastructure (CNKI), Library of Congress, and Web of Science. All potentially relevant articles in reference lists of included articles were screened as full-text. For missing information or ambiguous, the corresponding author of this study was contacted with authors of relevant articles by email. For duplicated duplications, we only included the latest articles in our analysis. More than 3,600 publications reporting the incidence of CT1DM were identified.

### Eligibility Criteria

Relevant studies for incidence of CT1DM in various countries were included in final analysis if the following strict criteria were met: (1) patients younger than 19 years old diagnosed with T1DM; (2) the number of cases was three or more; (3) the study period was more than a year; (4) T1DM was diagnosed according to World Health Organization definition. Studies met the following criteria were excluded: duplication (the same articles in different database); case reports, and comments; the studies not meeting criteria of inclusion. Eligibility assessment was independently conducted by 2 authors, with all inconsistent questions solved by discussion with other authors.

### Description of the data

Incidence data were extracted either from the text or from the tables in the publications. There was no incidence rate of the original articles were presented in the figures. Altogether 87 studies from 72 countries met the inclusion criteria and were finally included in this study (Table 1). In 78 studies the children aged from 0 to 14 years and in 9 studies from 0 to 12, 15, 17, 19 years. The time period of the researches ranged from 1 to 30 years. The degree of case-ascertainment ranged from 85 to 100%. The researches included in this study were from the period 1965 to 2012.

**Table 1:** The characteristics of worldwide incidence (per 100,000 children/yr) of childhood type 1 diabetes mellitus.

Regions and Centers	Study periods	Age-group (years old)	Main climate type	Boy*		Girl*		Total		References
				case (n)	Incidence	case (n)	Incidence	case (n)	Incidence	
***Africa***										
**Algeria**										
Oran	1980–1989	0–14	①					505	8.1	44
	1990	0–14		9	4.4	14	7	23	5.7 (3.62–5.82)	4
	1979–1988	0–14					4.7			45
	1990–1999	0–14			7.7		9.6		8.6 (7.6–9.8)	46
**Libya**										
Benghazi	1981–1990	0–19	②	121	8.3 (6.9–10.0)	130	9.2 (7.7–11.0)	251	8.8 (7.8–10.0)	47
	1991–1999	0–14			7.8		10.3		9 (8.0–10.2)	46
**Mauritius**	1986–1990	0–14	③		1.8		2.4		2.1	48
	1990–1994	0–14		10	1.3	11	1.5	21	1.4 (0.83–2.07)	4
**Sudan**	1991–1995	0–14	②					534	10.1 (9.0–12.8)	49
Khartoum			②					196	31.8 (28.4–35.2)	50
Gezira	1990	0–14	②	17	5.6	12	4.4	29	5(3.74–6.54)	4
**Sultanate of Oman**	1993	0–14	②		3.23		1.99		2.45	51
	1994	0–14			2.91		1.95		2.62	51
**Tunisia**										
Beja	1990–1994	0–14	②	22	9	16	6.5	38	7.8 (5.47–10.68)	4
	1990–1999	0–14			8.4		6.9		7.7 (6.1–9.6)	46
Gafsa	1990–1994	0–14	②	31	10	22	7.5	53	8.8 (6.59–11.51)	4
	1990–1999	0–14			9.5		7.5		8.5 (6.9–10.3)	46
Kairoan	1991–1993	0–14	②		7.3		7.8		7.6 (5.6–10.0)	46
Monastir	1990–1994	0–14	②	15	4.7	16	5.2	31	4.9 (3.35–6.96)	4
	1990–1999	0–14			6.6		5.1		5.8 (4.6–7.3)	46
**Tanzania**										
Dar es Salaam	1982–1991	0–14	④		0.8		0.9	86	0.8	52
***Asia***										
**China**										
Beijing	1990–1994	0–14	⑤	38	0.7	52	1.1	90	0.9 (0.72–1.09)	4
	1995–2000	0–14			0.93 (0.65–1.22)		1.60 (1.42–1.78)		1.25 (1.07–1.43)	53
	2001–2005	0–14			1.37 (1.26–1.48)		2.07 (1.62–2.51)		1.70 (1.48–1.91)	53
	2006–2010	0–14			2.05 (1.45–2.63)		2.48 (1.81–3.15		2.25 (1.64–2.85)	53
Chang Chun	1990–1994	0–14	⑤	7	0.6	11	1.1	18	0.8 (0.49–1.30)	46
Changsha	1990–1994	0–14	⑤	10	0.6	7	0.2	17	0.2 (0.2–0.4)	46
Dalian	1990–1994	0–14	⑤	10	1.1	11	1.2	21	1.1 (0.7–1.7)	46
Guilin	1991–1994	0–14	⑤	2	0.6	3	1	5	0.8 (0.2–2.0)	46
Hainan	1990–1994	0–14	⑤	6	0.1	11	0.2	17	0.2 (0.1–0.2)	46
Harbin	1990–1996	0–14	⑤	18	0.6	17	0.6	35	0.6 (0.4–0.8)	46
Hong-Kong	1986–1990	0–14	⑤		1.5		2.4	22	2	54
	1990–1994	0–14		4	0.6	13	2.1	17	1.3 (0.77–2.17)	4
	1990–1995	0–14			0.6		1.9		1.3 (0.8–1.9)	46
	1997	0–14						218	1.4	55
Huhehot	1990–1994	0–14	⑥	10	1.1	6	0.7	16	0.9 (0.5–1.5)	46
Jilin	1990–1994	0–14	⑥	8	0.4	14	0.8	22	0.6 (0.4–0.9)	46
Jinan	1990–1995	0–14	⑤	12	0.5	11	0.4	23	0.4 (0.3–0.6)	46
Lanzhou	1991–1994	0–14	⑥	5	0.4	3	0.2	8	0.3 (0.1–0.5)	46
Nanjing	1990–1994	0–14	⑤	7	0.3	13	0.7	20	0.5 (0.3–0.8)	46
Nanning	1990–1994	0–14	⑤	4	0.7	10	0.7	14	0.7 (0.5–0.9)	46
Shanghai	1980–1991	0–14	⑤	35	0.55 (0.38–0.76)	40	0.67 (0.45–0.91)	75	0.61 (0.48–0.77)	56
	1989–1993	0–14		28	0.78 (0.52–1.12)	30	0.88 (0.59–1.25)	58	0.83 (0.61–1.04)	57
	1990–1994	0–14		24	0.4	23	0.5	47	0.5 (0.3–0.7)	46
	1997–2011	0–14		306	3.1 (2.8–3.4)	316	3.2 (2.8–3.5)	622	3.1 (2.9–3.3)	39
Sichuan	1990–1994	0–14	⑤	9	1.8	13	2.7	22	2.3 (1.4–3.3)	46
Tie Ling	1990–1994	0–14	⑤	5	0.2	3	0.1	8	0.2 (0.1–0.3)	46
Wuhan	1990–1994	0–14	⑤	13	5.2	9	3.8	22	4.5 (2.8–7.0)	46
Wulumuqi	1990–1994	0–14	⑥	5	0.9	4	0.8	9	0.8 (0.3–1.7)	46
Zhengzhou	1991–1994	0–14	⑤	2	0.2	8	1	10	0.6 (0.3–1.1)	46
Zunyi	1990–1995	0–14	⑤	1	0	2	0.1	3	0.1 (0.0–0.2)	46
**India**										
Karnataka		0–15	⑤		3.7		4			58
**Israel**	1975–1980	0–14	①		4.4		6.7	296	5.5	59
	1989–1990	0–14		64	4.4 (3.4–5.6)	92	6.7 (5.4–8.2)	156	5.5 (4.7–6.5)	59
	1990–1993	0–14			5.5		6.6		6 (5.4–6.7)	46
	1990–1993	0–17		201	7.0 (6.1–8.0)	206	7.6 (6.6–8.7)	407	7.3 (6.6–8.0)	60
	1990–1994	0–14		167	5.5	194	6.6	361	6.0 (5.42–6.67)	4
**Japan**	1986–1990	0–14	⑤	522	1.2 (1.1–1.3)	738	1.8 (1.7–1.9)	1260	1.5 (1.4–1.6)	61
Chiba	1990–1993	0–14	⑤	27	1.2	34	1.6	61	1.4 (1.1–1.8)	46
Hokkaido	1974–1986	0–14	⑤		1.3		2.1	283	1.7	62
	1990–1993	0–14		45	2.2	44	2.1	89	2.2 (1.7–2.6)	46
Okinawa	1990–1993	0–14	③	6	1	11	1.8	17	1.4 (0.8–2.2)	46
**Kuwait**	1992–1993	0–14	②	47	16.58 (12.2–22.1)	39	14.11(10.0–19.3)	86	15.36 (12.4–19.1)	63
	1992–1994	0–14		82	19.2	71	17.3	153	18.3 (15.5–21.4)	4
	1992–1999	0–14			21.7		22.9		22.3 (20.5–24.2)	46
**Pakistan**										
Karachi	1990	0–14	②	9	0.5	16	0.9	25	0.7 (0.44–0.99)	4
	1990–1999	0–14			0.4		0.5		0.5 (0.3–0.5)	46
**Republic of Korea**										
Seoul	1985–1988	0–14	⑤		0.6		0.8	71	0.7	64
	1990–1991	0–14	⑤		1.1		1.2		1.1 (0.9–10.4)	46
**Russia**										
Novosibirsk	1983–1989	0–14	⑥		4.6		4.9		4.7	65
	1990–1994	0–14		90	5.7	101	6.4	191	6.0 (5.18–6.94)	4
	1990–1999	0–14			6.8		7.1		6.9 (6.3–7.6)	46
**Saudi Arabia**	1986–1997	0–14	②	19	9.9 (5.4–17.7)	27	14.8 (8.9–23.9)	46	12.3 (8.4–17.9)	66
Al-Madinah	2004–2009	0–12	②	170	22.2 (19.1–25.7)	249	33.0 (29.1–37.3)	419	27.6 (25.0–37.3)	67
Eastern	1990–2007	0–14	②	195		243		438	27.52 (26.7–28.3)	68
***Europe***										
**Austria**	1979–1993	0–14	③						7.8	45
	1989–1990	0–14		107	7.9 (6.5–9.3)	98	7.5 (6.1–9.2)	205	7.7 (6.7–8.8)	59
	1990–1994	0–14		348	9.8	312	9.3	660	9.6 (8.84–10.31)	4
	1990–1999	0–14			10.3		9.5		9.9 (9.4–10.4)	46
	2000–2005	0–14		610	14.8 (13.6–16.0)	561	14.3 (13.2–15.5)	1171	14.6 (13.7–15.4)	69
**Belgium**										
Antwerpen	1989–1990	0–14	③	15	9.2 (5.2–15.3)	16	10.4 (5.9–16.9)	31	9.8 (6.7–13.9)	59
	1990–1994	0–14		44	10.5	51	12.8	95	11.6 (9.40–14.41)	4
	1990–1999	0–14			10.7		12.8		11.7 (10.2–13.5)	46
**Belarus**										
Gomel	1976–1999	0–14	⑥					433	4.6 (4.4–4.8)	70
**Bosnia and Herzegovina**										
Tuzla	1990–1998	0–14	⑥	22	3.39 (1.8–4.9)	21	3.37 (1.7–5.0)	43	3.38 (2.3–4.5)	71
**Bulgaria**										
Sofia	1987–1991	0–14	⑥						6.7	72
East	1974–1995	0–14	⑥						6.3	45
Varna	1990–1994	0–14	①	82	5.9	100	7.6	182	6.8 (5.80–7.83)	4
	1990–1999	0–14			7.9		8.3		8.1 (7.4–9.0)	46
West-Bulgaria	1990–1994	0–14	③	131	9.9	125	10	256	9.9 (8.71–11.21)	4
	1990–1999	0–14			11.6		9.8		10.7 (9.8–11.6)	46
**Croatia**	1995–2003	0–14	①	369	9.26 (8.30–10.21)	323	8.47 (7.54–9.41)	692	9.05 (8.38–9.72)	25
Zagreb	1988–1992	0–14	⑥		7.7		6.7	72	7.2	73
**Czech Republic**	1990–1997	0–14	③	814	10.0 (9.4–10.7)	790	10.2 (9.5–11.0)	1604	10.1 (9.6–10.6)	74
	1995–1999	0–14			12.6		12.7		12.7 (11.9–13.5)	46
	1990–2001	0–14						2644	11.4 (11.0–11.9)	75
**Denmark**										
3 countries	1989–1994	0–14	③	34	21.5 (14.9–30.1)	32	21.4 (14.7–30.3)	66	21.5 (16.6–27.3)	59
Four countries	1990–1994	0–14	③	96	16.4	81	14.5	177	15.5 (13.3–17.9)	4
	1990–1999	0–14			17.1		16.2		16.6 (14.9–18.4)	46
**Estonia**	1983–1990	0–14	③	149	6.3(5.3–7.4)	142	6.3 (5.3–7.5)	291	10.1 (8.9–11.4)	76
	1991–1998	0–14		153	6.7 (5.7–7.9)	157	7.2 (6.1–8.4)	210	12.3 (11.0–13.8)	76
	1990–1994	0–14		85	9.9	93	11.2	178	10.5 (9.05–12.20)	4
	1990–1999	0–14			12.6		10.9		11.7 (10.6–13.0)	46
**Finland**	1987–1992	0–14	③	1113	37.6 (35.5–39.9)	949	33.5 (31.5–35.8)	2062	35.7 (34.1–37.2)	77
	1983–1990	0–14		1447	35.9 (34.1–37.8)	1198	31.2 (29.5–33.0)	2645	34.6 (33.3–36.0)	76
	1987–1991	0–14						1728	35.4 (33.9–37.4)	78
	1991–1998	0–14		1654		1497		3151	40.8 (39.4–42.2)	76
	1990–1994	0–14		915	37	853	36	1768	36.5 (34.8–38.3)	4
	1990–1999	0–14			41.9		39.9		40.9 (39.6–42.2)	46
2 regions	1989–1990	0–14	③	84	47.0 (37.5–58.1)	67	38.8 (30.5–50.0)	151	42.9 (36.6–50.6)	59
**France**	1988	0–19	③	96	7.86 (6.63–9.09)	79	6.96 (5.76–8.16)	175	7.41 (6.55–8.27)	79
	1997	0–19		117	10.48 (6.13–11.83)	93	8.68 (7.39–9.97)	210	9.58 (8.64–10.52)	79
Four regions	1989–1990	0–14	③	134	7.8 (6.6–9.3)	127	7.8 (6.5–9.2)	261	7.8 (6.9–8.8)	59
	1990–1994	0–14		372	8.7	337	8.3	709	8.5 (7.9–9.1)	46
**FYR Macedonia**	1995–1999	0–14	⑥		4.9		3.5		4.2 (3.4–5.2)	46
**Germany**	1999–2003	0–14	③		19.9 (19.0–20.7)		18.9 (18.1–19.8)	12335	19.4 (18.7–20.1)	80
	2004–2008	0–14			23.5 (22.5–24.5)		22.4 (21.4–23.3)	13299	22.9 (24.6–28.0)	80
Düsseldorf	1995–1999	0–14	③		14.8		16.1		15.4 (13.8–17.2)	46
Baden-Württemberg	1990–1994	0–14	③	463	11	440	10.9	903	11.0 (10.3–11.7)	4
	1990–1999	0–14			12.7		12.6		12.6 (12.1–13.2)	46
	1987–2003	0–14							14.1 (13.7–14.6)	81
	1999–2003	0–14			17.4(16.6–18.1)		16.5 (15.9–17.2)	1492	17.0 (16.4–17.6)	80
	2004–2008	0–14			22.7 (21.9–23.6)		21.7 (20.8–22.5)	1832	25.4 (24.1–26.8)	80
North Rhine-Westphalia	1999–2003	0–14	③		21.8 (21.1–22.5)		20.8 (20.0–21.5)	3112	21.3 (20.7–21.9)	80
	2004–2008	0–14			25.0 (24.1–25.8)		23.8 (23.0–24.6)	3295	24.4 (23.8–25.0)	80
Saxony	1999–2003	0–14	③		15.8 (14.7–16.9)		15.0 (14.0–16.1)	411	17.7 (15.9–19.6)	80
	2004–2008	0–14			20.8 (19.4–22.2)		19.8 (18.5–21.1)	445	20.3 (19.1–25.5)	80
**Greece**	1992	0–14	①		6.7		6.5	137	6.6	82
Attica	1990–1994	0–14	①	149	10.2	124	9.1	273	9.7 (8.55–10.92)	4
	1990–1999	0–14			11		9		10 (9.2–10.9)	46
Athens region	1989–1990	0–14	①	72	10.9 (8.5–13.7)	50	7.7 (5.7–10.2)	122	9.3 (7.7–11.1)	59
Northen 5 regions	1989–1990	0–14	①	9	5.3 (2.4–10.1)	6	3.8 (1.4–8.2)	15	4.6 (2.6–7.5)	59
**Hungary**	1978–1987	0–14	⑥					1060	6.1 (4.7–7.3)	83
Eighteen countries	1989–1990	0–14	⑥	132	7.7 (6.4–9.1)	124	7.5 (6.3–9.0)	256	7.6 (6.7–8.6)	59
	1990–1994	0–14		337	8.7	360	9.6	697	9.1 (8.43–9.81)	4
	1990–1999	0–14			9.6		9.8		9.7 (9.2–10.2)	46
**Italy**	1990–2003	0–14	①	2840	13.13 (12.66–13.62)	2340	11.35 (10.90–11.82)	5180	12.26 (11.93–12.60)	84
Lazio	1989–1990	0–14	①	66	7.2 (5.5–9.2)	51	5.8 (4.4–7.7)	117	6.5 (5.4–7.8)	59
	1990–1994	0–14		164	8	162	8.3	326	8.1 (7.28–9.07)	4
	1990–1999	0–14			8.9		8.6		8.8 (8.1–9.4)	46
Lombardia	1989–1990	0–14	①	110	7.6 (6.3–9.2)	83	5.9 (4.7–7.3)	193	6.8 (5.8–7.8)	59
	1990–1994	0–14		239	7.6	204	6.8	443	7.2 (6.55–7.92)	4
	1990–1995	0–14			7.2		6.5		6.9 (6.3–7.5)	46
Marche	1990–1994	0–14	①	55	10.5	44	8.9	99	9.7 (7.90–11.84)	4
	1990–1999	0–14			10.5		9.7		10.1 (8.8–11.6)	46
Pavia	1990–1994	0–14	①	17	11.6	17	11.9	34	11.7 (8.08–16.44)	4
	1990–1999	0–14			12.3		12.5		12.4 (9.7–15.6)	46
Sardinia	1989–1990	0–14	①	126	33.5 (27.9–39.9)	95	26.9 (21.7–32.9)	221	30.2 (26.4–34.4)	59
	1990–1994	0–14		337	43.6	211	29.5	548	36.8 (33.72–39.98)	4
	1990–1998	0–14			45		30.6		37.8 (35.5–40.3)	46
Eastern Sicily	1989–1990	0–14	①	29	11.2 (7.5–16.1)	23	9.0 (5.7–13.5)	52	10.1 (7.5–13.2)	59
	1990–1994	0–14		75	13.4	53	9.9	128	11.7 (9.8–13.9)	46
Turin	1984–1991	0–14	①	116	8.42 (6.99–10.10)	111	8.42 (6.95–10.19)	227	8.42 (7.37–9.62)	85
	1990–1994	0–14		86	11.9	69	10.1	155	11.0 (9.32–11.15)	4
	1990–1999	0–14			11.7		10.3		11 (9.8–12.3)	46
Roman and Lazio region	1989–1993	0–14	①		7.9(6.8–9.2)		7.8(6.7–9.1)		7.9 (7.1–8.8)	86
Liguria	1989–1998	0–14	①	126	14.15 (11.9–16.9)	93	10.88 (8.9–13.3)	219	12.56 (11.0–14.3)	87
**Iceland**	1970–1979	0–14	③	31	9.3 (6.3–13.2)	21	6.6 (4.1–10.1)	52	8.0 (8.4–13.8)	88
	1980–1989	0–14		34	10.5 (7.3–14.7)	34	11.1 (7.6–15.5)	68	10.8 (8.4–13.8)	88
**Latvia**	1983–1990	0–14	③	229	6.6 (5.8–7.5)	227	6.7 (5.8–7.6)	456	6.6 (5.8–7.3)	76
	1991–1998	0–14		242	6.9 (6.0–7.8)	263	7.7 (6.8–8.7)	505	7.4 (6.6–8.2)	76
	1990–1992	0–14		59	7	47	5.7	106	5.9 (5.06–6.98)	4
	1990–1999	0–14			7.8		7		7.4 (6.6–8.3)	46
**Lithuania**	1983–1990	0–14	③	143	9.7 (8.2–11.4)	132	9.5 (7.9–11.3)	275	6.8 (6.2–7.5)	76
	1991–1998	0–14		162	12.5 (10.7–14.6)	139	10.9 (9.1–12.8)	301	7.8 (7.1–8.5)	76
	1990–1994	0–14		162	7.7	145	7.1	307	7.4 (6.57–8.25)	4
	1990–1999	0–14			7.6		8.2		7.9 (7.3–8.5)	46
	1983–2000	0–14		543	7.3 (6.7–7.9)	557	7.8 (7.1–8.4)	1100	7.5 (7.1–8.0)	89
**Luxemburg**	1977–1986	0–14	③		12.1		12.6	16	12.4	59
	1989–1990	0–14		8	12.1 (5.2–23.9)	8	12.6 (5.4–24.8)	16	12.4 (7.1–20.1)	59
	1990–1994	0–14		22	12.6	17	10.2	39	11.4 (8.14–15.59)	4
	1990–1999	0–14			10.3		12.2		11.3 (9.0–13.9)	46
**The Netherlands**										
Five regions	1989–1990	0–14	③	30	11.2 (7.6–16.0)	28	10.8 (7.2–15.7)	58	11.0 (8.4–14.3)	59
	1990–1994	0–14		178	12.9	175	13.2	353	13 (11.7–14.4)	46
**Macedonia**	1985–1991	0–14			2.4		2.5	112	2.5	90
**Malta**	1980–1987	0–14	⑥	43	12.7 (9.6–15.8)	47	14.6 (11.3–17.9)	90	13.6 (11.0–16.2)	91
	2006–2010	0–14		41		40		81	24.68 (21.98–27.43)	92
**Montenegro**	1997–2006	0–14	③	90	12.6 (10.1–15.5)	94	14.3 (11.5–17.5)	184	13.4 (11.5–15.5)	93
**Norway**	1973–1982	0–14	③					1914	20.5	94
	2004–2012	0–14			33.9 (32.2–35.7)		31.4 (29.7–33.2)		32.7 (31.5–34.0)	95
Eight countries	1989–1990	0–14		87	22.3 (17.9–27.6)	71	19.3 (15.1–24.3)	158	20.8 (17.7–24.3)	59
	1990–1994	0–14		222	22.4	187	19.9	409	21.2 (19.18–23.29)	4
	1990–1999	0–14			21.6		19.9		20.8 (19.4–22.1)	46
**Poland**										
9 western provinces	1989–1990	0–14	⑥	80	5.3 (4.6–6.5)	84	5.8 (4.6–7.2)	164	5.5 (4.7–6.4)	59
3 cities	1989–1990	0–14	⑥	51	5.7 (4.2–7.5)	51	6.0 (4.5–7.9)	102	5.8 (4.8–7.1)	59
Cracow	1990–1999	0–14	⑥		7.5		7.6		7.6 (7.0–8.2)	46
Upper Silesia	1995–1999	0–14	⑥		8		9.5		8.8 (7.9–9.7)	46
Wielkopolska	1990	0–14	⑥	28	4.1	40	6	68	5 (3.88–6.36)	4
**Portugal**										
3 regions combined	1989–1990	0–14	①	17	10.1 (5.9–16.1)	8	4.9 (2.1–9.6)	25	7.5 (4.8–11.0)	59
Algarve	1990–1994	0–14	①	26	16.3	19	12.9	45	14.6 (10.62–19.64)	4
Coimbra	1990–1994	0–14	①	19	9.4	19	9.9	38	9.7 (6.76–13.36)	4
	1990–1999	0–14			10.1		9.1		9.6 (7.6–12.2)	46
Madeira Island	1990–1994	0–14	③	10	6.9	11	7.5	21	7.2 (4.46–11.05)	4
	1990–1999	0–14			7.1		6.8		6.9 (5.0–9.4)	46
Portalegre	1990–1994	0–14	①	9	15.9	14	26.7	23	21.3 (13.29–31.89)	4
**Romania**										
Bucharest	1989–1990	0–14	⑥	22	4.6 (2.9–6.9)	25	5.7 (3.7–8.4)	47	5.1 (3.8–6.8)	59
	1990–1994	0–14		52	4.2	65	5.9	117	5.0 (4.14–6.05)	4
	1990–1999	0–14			4.7		5.9		5.3 (4.7–6.1)	46
**Silesian**	1989–2005	0–14	⑥	720	10.01 (8.58–11.45)	665	9.72 (8.32–11.31)	1385	9.87 (8.45–11.47)	96
**Slovakia**	1990–1994	0–14	⑥	261	7.9	289	9.1	550	8.5 (7.81–9.25)	4
	1990–1999	0–14			9.7		9.7		9.7 (9.2–10.3)	46
	2000	0–14		81	15.04	66	12.83	147	13.96 (11.35–15.72)	97
**Slovenia**	1989–1990	0–14	①	23	5.2 (3.3–10.4)	33	7.7 (5.3–10.9)	56	6.5 (4.9–8.4)	59
	1990–1994	0–14		70	6.8	88	9	158	7.9 (6.68–9.23)	4
	1990–1998	0–14		142	8.28 (6.9–9.6)	157	9.63 (8.1–11.1)	299	8.94 (7.9–9.9)	71
	1990–1999	0–14			8.3		9.5		8.9 (8.0–9.9)	46
**Spain**										
Catalonia	1989–1990	0–14	①	151	10.5 (8.8–12.3)	146	10.6 (9.0–12.5)	297	10.6 (9.4–11.9)	59
	1990–1994	0–14		358	12.5	338	12.6	696	12.5 (11.55–13.50)	4
	1990–1999	0–14			12.6		12.3		12.4 (11.7–13.1)	46
Biscay	1990–2013	0–14	③	199	10.4 (8.9–11.8)	200	11.5 (9.5–12.6)	399	10.7 (9.6–11.7)	98
Extremadura	2003–2007	0–14	①	104	24.9 (20.1–29.7)	104	26.2 (21.2–31.6)	208	25.5 (22.1–29.0)	99
Madrid	1985–1988	0–14	⑥		11.3		10.5	501	10.9	100
Cordoba	1991–1992	0–14	①	21	6.2	26	7.9	47	7 (5.20–9.26)	4
**Sweden**	1978–1987	0–14	③	2012	25	1824	23.8	3838	24.4	101
	1990–1994	0–14		1135	28.1	1031	26.9	2166	27.5 (26.36–28.67)	4
	1990–1999	0–14			30.5		29.4		30 (29.1–30.8)	46
	1983–2000	0–14		4171	29.2 (28.3–30.1)	3860	28.5 (27.6–29.4)	8031	28.9 (28.2–29.5)	89
	2002–2004	0–14			42.9 (38.7–47.7)		42.1 (37.6–46.7)	2046	42.5 (39.3–45.7)	102
	2005–2007	0–14			46.7 (41.6–51.5)		41.2 (36.0–45.6)	2029	43.9 (40.7–47.3)	102
**Serbia**										
Belgrade	1982–1992	0–14	⑥	126	7.6 (6.4–9.1)	133	8.6 (7.2–10.2)	289	8.1 (7.1–9.2)	103
	1982–2005	0–14		372	10.6 (9.5–11.8)	330	10.5 (9.4–11.7)	702	10.6 (9.8–11.4)	104
**Switzerland**	1995–1999	0–14	①		13.3		10.7		12 (11.2–12.9)	46
**Turkey**										
Diyarbakir	2010–2011	0–14	⑥	24	8.7	17	5.7	41	7.2	105
**UK**										
Scotland	1976–1983	0–14	③		20		19.4	1856	19.7	62
	1990	0–14		16	32.5	7	15	23	24.0 (15.22–36.01)	4
	1990–1999	0–14			26.8		25.9		26.4 (25.4–27.4)	46
Leicestershire	1971–1980	0–14							10.6 (5.1–17.1)	106
	1990–1994	0–14	③	70	15.4	66	15.3	136	15.3 (12.85–18.07)	4
Northern Ireland	1989–1990	0–14	③	71	17.8 (13.9–22.5)	59	15.4 (11.7–19.8)	130	16.6 (13.9–19.7)	59
	1990–1994	0–14		202	20.1	185	19.3	387	19.7 (17.81–21.79)	4
	1990–1999	0–14			21.5		21.2		21.3 (19.9–22.8)	46
Oxford	1985–1995	0–14	③	572	19.9 (18.3–21.5)	465	17.2 (15.6–18.7)	1037	18.6 (17.4–19.8)	107
	1989–1990	0–14		90	17.8 (14.3–21.9)	71	14.9 (11.7–18.8)	161	16.4 (13.9–19.1)	59
	1990–1994	0–14		266	20.1	191	15.3	457	17.8 (16.18–19.46)	4
Plymouth	1990–1994	0–14	③	63	16.5	65	18.1	128	17.3 (14.41–20.53)	4
	1990–1999	0–14			17.1		20.8		19 (16.8–21.2)	46
Yorkshire	1978–2007	0–14	③					2662	18.1 (17.6–18.7)	108
	1990–1999	0–14			18.9		18.1		18.5 (17.5–19.5)	46
Tayside	1980–1983	0–14	③		19.7		22.1	64	20	62
Bradford	1978–1998	0–14	③	142	12.4 (10.4–14.4)	147	13.6 (11.4–15.8)	289	13.0 (11.5–14.5)	109
Far the south-west England	1975–1996	0–14	③	228	13.63 (12.00–15.47)	260	16.29 (14.49–18.38)	488	14.93 (13.58–16.16)	110
***North America***										
**Canada**										
Newfoundland and Labrador	1995–2002	0–19	⑥	400	77.3 (69.9–85.3)	494	100.2 (91.6–109.4)	894	88.6 (74.0–105.4)	111
Edmonton	1990–1996	0–14	⑥		23		23.6		23.3 (20.5–26.4)	46
Calgary	1990–1999	0–14	⑥		20.3		20.9		20.6 (18.5–22.7)	46
Prince Edward Island	1975–1986	0–14	⑥		27		20.8	92	23.9	62
	1990–1993	0–14		17	28	12	20.8	29	24.5 (16.38–35.16)	4
The Avalon Peninsula	1987–2002	0–14	⑥	140	36.15	134	35.69	274	35.93	112
Montreal	1971–1985	0–14	⑥		9.6		10	919	9.8	62
	1971–1983	0–14			9		9.1		9.0 (7.7–10.6)	113
Alberta	1990–1994	0–14	⑥	87	23.4	88	24.7	175	24.0 (20.62–27.82)	4
Manitoba	1991–1993	0–14	⑥		21.4		20.7		21.1 (17.1–25.9)	4
**USA**										
Allegheny, PA	1990–1994	0–14	⑥	112	19.1	94	16.4	206	17.8 (15.45–20.33)	4
Chicago, IL	190–1994	0–14	⑥	131	10.2	169	13.3	300	11.7 (10.47–13.12)	4
	1994–2003	0–17		617	16.0 (14.6–17.6)	749	20.1 (18.3–22.1)	1366	18.1 (16.9–19.3)	114
Jefferson, AL	1990–1994	0–14	⑥	50	14.6	51	15.4	101	15.0 (12.21–18.22)	4
	1990–1995	0–14			14.1		15.1		14.6 (12.2–18.2)	46
Colorado			⑥							
non-Hispanics	1978–1988	0–17		654	16.4 (15.1–17.7)	544	14.5 (13.3–15.7)			115
Hispanics	1978–1988	0–17		56	7.1 (5.4–9.3)	79	10.5 (8.4–13.1)			115
North Dakota	1980–1986	0–14	⑥		21.6		16.2	204	18.9	116
Wisconsin (part)	1970–1979	0–14	⑥		20.2		16.2	166	18.2	62
Rochester	1965–1979	0–14	⑥		15.8		18.4	38	17.1	62
Philadelphia	1985–1989	0–14	⑥		11.3		14.8	215	13.4	117
San Diego	1978–1981	0–14	①		9.6		9.1	48	9.4	62
***South America***										
**Argentina**										
Avellaneda	1985–1990	0–14	④					30	6.7	118
	1990–1994	0–14		11	5.6	15	7.5	26	6.5 (4.31–9.51)	4
	1990–1996	0–14			5.3		7.2		6.3 (5.7–11.1)	46
Corrientes	1992–1994	0–14	④	4	2.9	8	5.7	12	4.3 (2.21–7.51)	4
	1992–1999	0–14			4.7		8.5		6.6 (5.0–8.7)	46
Tierra del Fuego	1993–1994	0–14	③	4	20.2	0		4	8.0 (2.18–17.60)	4
	1993–1996	0–14			14.2		6.3		10.3 (5.5–18.5)	46
**Brazil**										
Sao Paulo	1987–1991	0–14	⑤		5.8		9.5	52	7.6	119
	1990–1992	0–14		15	6.9	19	9.1	34	8 (5.53–1.14)	4
Passo Fundo	1996–1999	0–14	⑤		5.4		8.7		7 (4.1–11.9)	46
**Chile**	1990–1991	0–14	②		2.2		2.8	52	2.5	120
Santiago	1990–1992	0–14	①	66	1.7	56	1.5	122	1.6 (1.28–2.04)	4
	1990–1999	0–14			3.6		3.9		3.7 (3.4–4.0)	46
**Colombia**	1990	0–14	⑦		4.7		2.9		3.8 (2.9–4.9)	4
Cali	1995–1999	0–14	⑦		0.4		0.5		0.5 (0.3–0.7)	46
Bogota	1990	0–14	⑦	35	4.7	21	2.9	56	3.8 (2.88–4.93)	4
**Paraguay**	1990–1994	0–14	④	45	1	34	0.8	79	0.9 (0.71–1.11)	4
	1990–1999	0–14			1		0.8		0.9 (0.8–1.0)	46
**Peru**										
Lima	1990–1991	0–14	②		0.2		0.6		0.4 (0.22–0.61)	4
	1990–1994	0–14			0.4		0.6		0.5 (0.4–0.64)	46
**Uruguay**										
Montevideo	1992	0–14	⑤	13	8.3	13	8.3	26	8.3 (5.38–12.10)	4
**Venezuela**										
Caracas	1992	0–14	④	18	0.1	25	0.2	43	0.1 (0.09–0.18)	4
***Central America and West Indies***										
**Antigua**	1989–1993	0–19	③						3.5 (0.9–8.8)	121
**Barbados**	1982–1991	0–14	③					37	5	122
	1989–1993	0–19							2.6 (1.3–4.6)	121
	1990–1993	0–14		3	2.4	2	1.6	5	2.0 (0.32–6.36)	4
**Cuba**	1978–1990	0–14	⑦		2.5		2.8	267	2.7	62
	1990–1994	0–14		152	2.5	197	3.4	349	2.9 (2.63–3.24)	4
	1990–1999	0–14			2.1		2.5		2.3 (2.2–2.5)	46
**Dominican Republic**	1990–1993	0–14	③	3	6.6	2	4.9	5	5.7 (1.53–14.65)	4
	1995–1999	0–14			0.7		0.3		0.5 (0.4–0.7)	46
**Mexico**										
Mexico city	1984–1986	0–14	⑥		0.4		0.7	100	0.6	62
Veracruz	1990–1993	0–14	④	3		6		9	1.5 (0.70–2.94)	4
**Puerto Rico (USA)**	1985–1994	0–14	⑦						18.0 (17.6–18.3)	123
	1990–1994	0–14		398	16.2	445	18.7	843	17.4 (16.25–18.63)	4
	1990–1999	0–14			15.8		17.8		16.8 (16.0–17.6)	46
**Virgin Islands (USA)**	1990–1994	0–14	③	9	14.7	7	11.5	16	13.1 (7.64–21.01)	4
	1990–1996	0–14					14		12.8 (8.1–18.8)	46
***Oceania***										
**Australia**	2000–2011	0–14	②	6049	24.2 (23.6–24.8)	5526	23.0 (22.4–23.7)	11575	23.6 (23.2–24.0)	124
West	1985–1992	0–14	④						14.9	45
	1985–2002	0–14		560	15.6 (13.7–17.5)	584	17.3 (15.3–19.4)	1144	16.5 (14.7–18.2)	24
	1985–2010	0–14			17.7 (16.9–19.3)		18.5 (17.4–19.8)		18.1 (17.5–19.2)	125
New South Wales	1990–1993	0–14	①	335	13.1	387	15.9	722	14.5 (13.42–15.55)	4
	1990–1996	0–14			17.0 (14.1–20.6)		18.6 (15.4–22.3)		17.8 (15.6–20.3)	26
**New Zealand**										
Auckland	1978–1985	0–14	③		9		10.5	233	9.8	62
	1990–1994	0–14		65	12.3	70	13.6	135	12.9 (10.87–15.28)	4
	1990–1996	0–14			12.9		14.6		13.7 (12.0–15.7)	46
Canterbury	1981–1986	0–14	③		10.2		12.9	39	11.6	62
	1990–1994	0–14		43	23.9	35	19.8	78	21.9 (17.33–27.32)	4
	1990–1999	0–14			23.8		20.8		22.3 (19.1–25.9)	46

Data showed as mean (95% CI); *represented boy vs. girl, *p* > 0.05, *p* derived from t-test; ①, Mediterranean climate; ②, Desert Climate; ③, Oceanic climate; ④, Savanna climate; ⑤, Monsoon climate; ⑥, Continental climate; ⑦, Rainforest climate.

### Quality assessment

All abstracts ascertained initial search were screened and the researches in violation of inclusion criteria were excluded by two authors. Full-texts were posteriorly accessed by another two authors, in case of disagreement, a third professor was invited to evaluate such studies and the consensus was achieved via discussion. If original data was missing, the corresponding author of this study was contacted with alone tailored application forms by email.

### Climate Style, latitude, and sunshine durations

Mediterranean climate, monsoon climate, oceanic climate, continental climate, savanna climate and rainforest climate were included in this study. Climate style met the announcement of national climate center, and the missing information was searched in the climate of the countries of the world^19^. Latitude of every center was identified by Google Earth’s high-resolution satellite image^20^, and if the countries didn’t have centers records, we would extract the latitude of the capital. Sunshine durations of the capital in each country was ascertained by average sunshine durations timetable around the world^21^. Mediterranean climate is the climate typical of the lands around the Mediterranean Sea from the largest areas, but it is also found in sections of Asia, in most coastal California, and in parts of Southern and West of Australia. Monsoon is currently defined as a seasonal changing in atmospheric precipitation and circulation associated with the asymmetric heating of land and sea. Oceanic climate is the typical of west coasts in higher middle latitudes of regions, with few extremes of temperature and a relatively narrow annual temperature range, and generally features cool summers and winters. Continental climate is referred to climates with significantly annual variation in temperature, which tended to occur in the middles of continents, mostly occur in the mainland China and the eastern U.S.^22^.

### Statistical methods

The incidence of CT1DM for our study was obtained from the individual studies as it was researched in these publications. The incidence rates were calculated per 100, 000 people a year. Age standardization of the incidence rates was calculated using 5-years intervals with age groups 0–4 years, 5–9 years, and 10–14 years as the standard. The latitude groups 0°–23°26′N/S, 23°26′–40°N/S, and 40°–66°34′N/S as the study standard according to the tropic of Cancer/Capricorn, the Arctic/Antarctic circle, and westerlies, which based on geographic meteorology.

Statistical analysis was performed using SPSS version 20.0 (SPSS Inc., Chicago, IL, USA). Continuous data that accord with a normal distribution were presented as mean [95% confidence interval (CI)], with least significant difference in parameters between two groups were analyzed by *t*-test, and the one-way ANOVA was used to assess the multiple groups for continuous variables in normal distribution. A *p* < 0.05 is considered to be statistically significant difference.

## Results

### Description of the included studies

After initial screening and removal of duplicates, we reviewed 3602 articles in full, of which 87 eligible studies on the incidence of CT1DM in various countries were included in this study (Table 1). Included studies on incidence of CT1DM entailed 118 records for centers in 72 countries. The numbers of records were available for North America (n = 17), South America (n = 10), Asia (n = 30), Europe (n = 47), Oceania (n = 3), Central America and West Indies (n = 2), and Africa (n = 9). The numbers of records were obtainable for Mediterranean climate (n = 22), Monsoon climate (n = 22), Oceanic climate (n = 22), Continental climate (n = 34), Desert climate (n = 11), Savanna climate (n = 5), and Rainforest climate (n = 2). The specific characteristics of included articles are displayed in Tables 1 and 2.

**Table 2:** The characteristics of incidence of childhood type 1 diabetes mellitus (per 100,000 children/yr) in different age-groups.

Countries and centers	Search periods	0–4 years old*		5–9 years old**		10–14 years old	
		Case (n)	Incidence	Case (n)	Incidence	Case (n)	Incidence
**Australia**	2000–2011	2402	14.9 (14.3–15.5)	4007	24.7 (23.9–25.4)	5166	31.0 (30.2–31.9)
West	1985–2002	249	11.0 (9.2–12.8)	437	18.8 (16.3–21.3)	458	19.6 (17.6–21.6)
	1985–2010		11.0 (10.3–12.6)		21.1 (19.5–22.6)		25.5 (20.8–23.9)
New South Wales	1990–1996		10.8 (7.9–14.4)		17.8 (14.1–22.4)		25.0 (20.4–30.5)
**Belarus**	1976–1999		2.7		5.2		9.3
**Canada**							
Newfoundland and Labrador	1995–2002	59	29.6 (22.6–38.3)	213	90.5 (78.9–103.6)	348	127.4 (114.4–141.5)
The Avalon Peninsula	1987–2002	58	24.95	95	37.01	121	43.62
**China**							
Shanghai	1980–1991	16	0.26 (0.15–0.42)	41	1.25 (0.89–1.70)	18	0.62 (0.37–0.98)
	1989–1993	15	0.56 (0.32–0.93)	28	1.02 (0.68–1.47)	15	0.94 (0.52–1.55)
Hong Kong	1997	43	0.9	84	1.5	91	1.7
Beijing	1995–2000		0.41 (0.20–0.61)		1.47 (1.07–1.90)		1.49 (1.21–1.73)
	2001–2005		0.79 (0.65–0.93)		1.79 (1.43–2.15)		2.22 (1.91–2.53)
	2006–2010		0.92 (1.81–3.15)		2.83 (1.68–3.85)		2.99 (1.93–4.04)
**Croatia**	1995–2003	134	5.77 (4.79–6.74)	255	9.80 (8.60–11.01)	303	11.13 (9.88–12.38)
**Czech**	1990–1997		5.9 (5.3–6.7)		10.5 (9.7–11.5)		13.1 (12.2–14.1)
**Germany**							
	1999–2003		14.5 (14.0–15.1)		21.5 (20.1–22.9)		22.2 (20.8–23.7)
	2004–2008		17.1 (16.5–17.8)		25.4 (23.8–27.1)		26.3 (24.6–28.0)
Baden-Württemberg	1987–2003		5.8 (2.5–9.3)		3.4 (0.8–6.0)		2.7 (0.3–5.1)
	1999–2003		12.7 (11.9–13.5)		18.8 (17.7–19.9)		19.4 (18.3–20.6)
	2004–2008		16.6 (15.6–17.6)		24.6 (23.3–25.9)		25.4 (24.1–26.8)
North Rhine-Westphalia	1999–2003		15.9 (15.1–16.8)		23.5 (22.5–24.6)		24.4 (23.3–25.5)
	2004–2008		18.2 (17.3–19.2)		27.0 (25.8–28.2)		28.0 (26.8–29.2)
Saxony	1999–2003		11.5 (10.3–12.8)		17.0 (15.3–18.9)		17.7 (15.9–19.6)
	2004–2008		15.2 (13.7–16.8)		22.4 (20.3–24.8)		23.2 (21.0–25.7)
**Italy**							
Rome and Lazio Region	1989–1993	78	0.3 (5.0–7.9)	130	9.8 (8.3–11.6)	122	7.5 (6.2–9.0)
Turin	1984–1991	40	5.49 (3.92–7.47)	62	7.30 (5.69–9.49)	125	11.17 (9.49–13.49)
Liguria	1989–1998	50	9.01 (6.7–11.9)	72	13.03 (10.2–16.4)	97	15.01 (12.2–18.3)
Apulia	2009–2013	149	20.1 (16.8–23.3) 1–4years	296	29.7 (26.3–33.1)	299	28.2 (25.0–31.4)
**Jordanian**	19992–1996	39	1.3	90	3.2	146	5.5
**Kuwait**	1992–1993	27	12.83 (8.46–18.74)	30	15.71 (10.60–22.46)	29	18.29 (12.25–26.34)
**Libya**							
Benghazi	1981–1990	21	2.2 (1.4–3.4)	54	7.2 (5.3–9.5)	90	14.8 (12.0–18.4)
Lithuania	1983–2000	185	4.0 (3.5–4.6)	395	8.0 (7.2–8.8)	520	10.5 (9.6–11.5)
**Saudi Arabia**	1986–1997	8	7.1 (3.6–13.2)	13	7.1 (3.7–13.2)	25	24.1 (15.9–35.7)
Ai-Madinah	2004–2009	115	17.1 (14.2–20.5)	178	30.9 (26.6–35.7)	126	46.5 (38.9–55.2)
**Serbia**							
Belgrade	1982–1992	40	3.9 (2.8–5.3)	98	8.9 (7.3–10.9)	121	11.2 (9.3–13.4)
	1982–2005	108	5.5 (4.5–6.7)	256	11.9 (10.5–13.5)	346	15.4 (13.8–17.1)
**Silesian**	1989–2005		5.33 (4.31–6.55)		9.86 (8.45–11.45)		13.20 (11.53–15.05)
**Slovenia**	1990–1998	59	6.17 (4.5–7.7)	103	9.20 (7.4–10.9)	137	10.79 (9.0–12.6)
**Bosnia and Herzegovina**							
Tuzla	1990–1998	3	0.80 (0–1.7)	18	4.68 (2.5–6.8)	22	5.16 (2.8–7.5)
**Slovakia**	2000		10.5		12.57		17.97
**Spain**							
Extremadure	2003–2007	48	18.5 (10.1–30.3)	66	25.2 (20.1–29.4)	94	31.8 (25.8–34.1)
Biscay	1990–2013	57	5.1 (3.8–6.5)	168	14.6 (12.4–16.8)	174	13.2 (11.3–15.2)
**Sultanate of Oman**	1993		1.54		2.32		3.69
	1994		0.97		2.79		4.22
**Sweden**	1978–1987	759	15.7	1345	25.8	1734	30.6
	1983–2000	1816	19.5 (18.6–20.4)	2961	31.7 (30.6–32.8)	3254	35.4 (34.2–36.6)
	2002–2004	408	28.7 (23.9–33.5)	765	50.9 (44.5–57.0)	873	46.7 (41.5–52.2)
	2005–2007	387	25.2 (20.8–29.6)	676	47.9 (41.6–54.1)	966	56.5 (50.5–62.9)
**Turkey**							
Diyarbakir	2010–2011	8	4.3	17	9.1	16	8.4
**UK**							
Leicestershire	1971–1980		6.3 (1.3–8.9)		10.9 (1.6–19.5)		15.1 (5.9–23.7)
Yorkshire	1978–2007	807	11.7 (10.9–12.5)	1330	18.6 (17.6–19.6)	1774	23.7 (22.6–24.8)
Bradford	1978–1998	70	9.3 (7.1–11.5)	88	12.1 (9.6–14.7)	131	17.9 (14.9–21.0)
Far the south-west	1975–1996	96	9.35 (7.57–11.42)	170	15.81 (13.52–18.37)	222	19.02 (16.44–21.51)
**USA**							
Chicago	1994–2003	178	8.1 (7.0–9.5)	340	15.3 (13.7–17.2)	560	28.1 (25.5–30.9)

Data showed as mean (95% CI); *represented 0–4 years old vs. 5–9 years old, p > 0.05; 0–4 years old vs. 10–14 years old, p < 0.01; **represented 5–9 years old vs. 10–14 years old, p > 0.05, *p* derived from one-way ANOVA.

### Incidence of CT1DM

**Figure 1:**
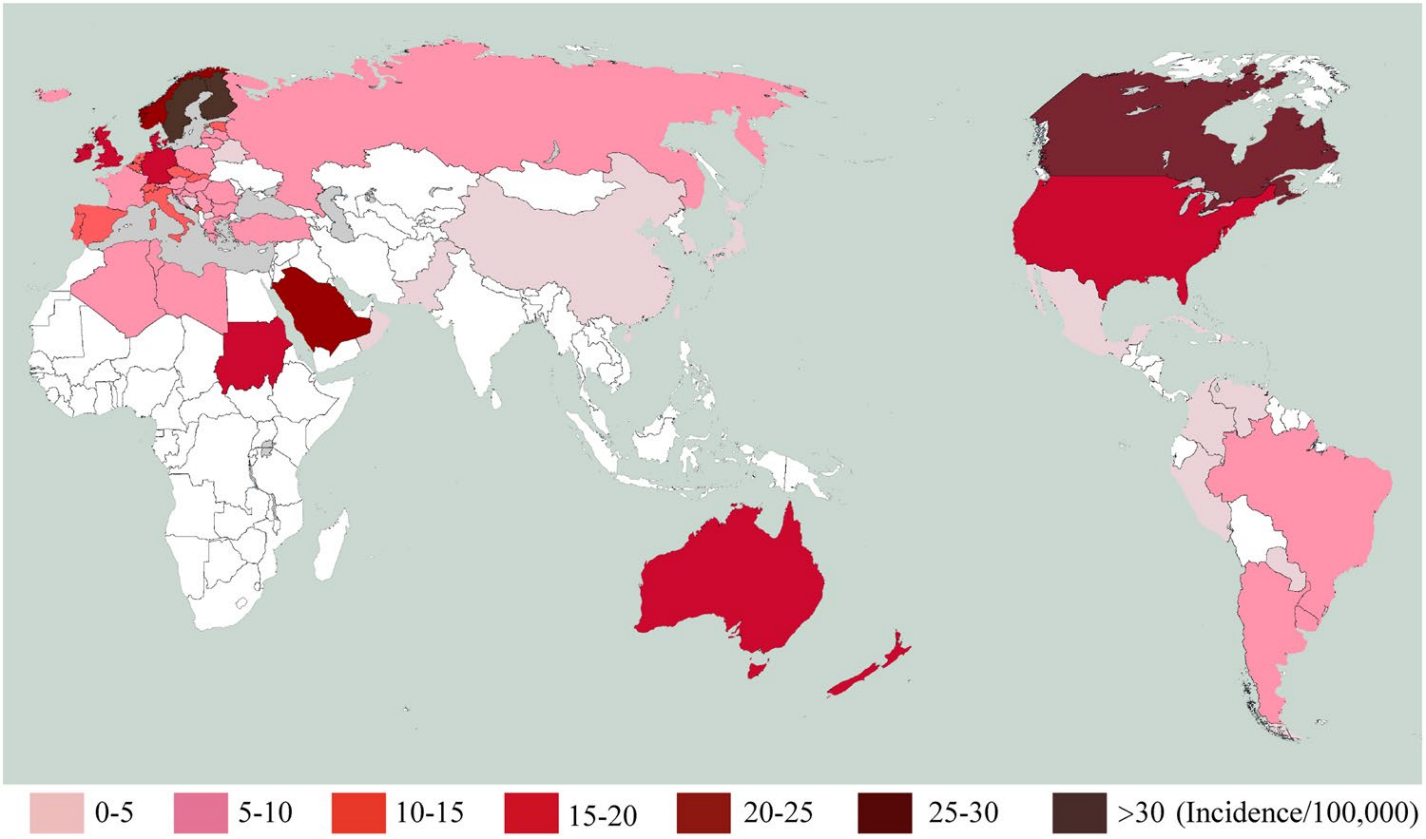
Incidence of childhood type 1 diabetes mellitus in 72 countries (the first author independently created map by software-Adobe Illustrator CS5 and Adobe Photoshop CS5, and the copyright of map belongs to first author).

The average incidence of CT1DM in 70 countries showed in Fig. 1.

#### Overall incidence of CT1DM

Overall incidence of CT1DM was 11.43 (10.31–12.55) per 100,000 children/yr, in addition, boy, 11.42 (10.23–12.61) per 100,000 children/yr; girl, 11.11 (9.94–12.27) per 100,000 children/yr. There no significant difference existed between two groups of gender (*p* > 0.05) (Table 1).

#### Incidence of CT1DM in different regions

**Figure 2:**
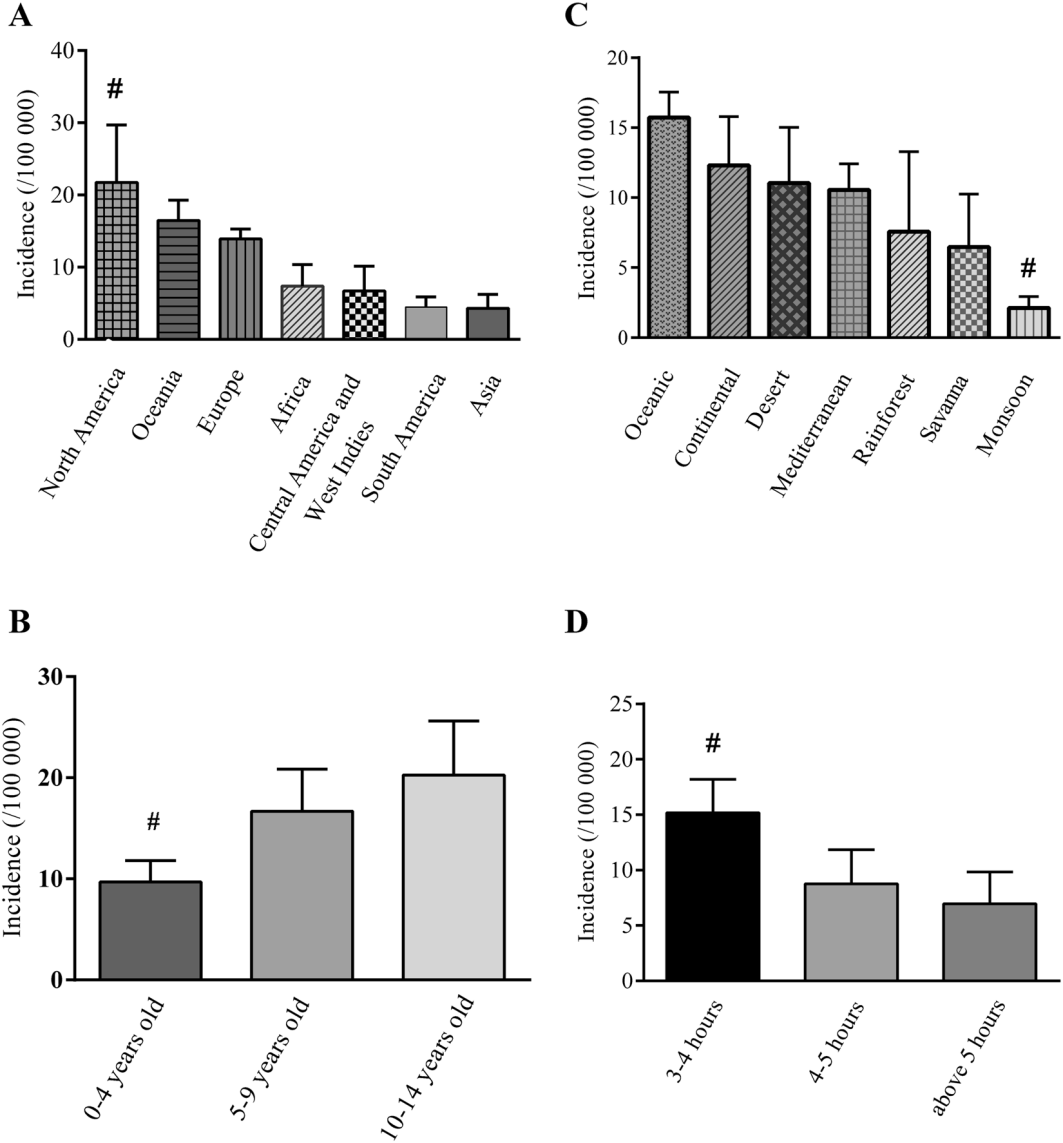
Incidence of childhood type 1 diabetes mellitus in different regions, age-groups, climates, and sunshine durations. (**A**, Incidence of childhood type 1 diabetes mellitus in different regions: ^#^indicated North America vs. other regions excluded Oceania, all *p* < 0.01; **B**, Incidence of childhood type 1 diabetes mellitus in three age-groups: ^#^represented 0–4 years old vs. 10–14 years old, *p* < 0.01; **C**, Incidence of childhood type 1 diabetes mellitus in seven kinds of climates: *represented Monsoon climate vs. other climates excluded Savanna climate and Rainforest climate, all *p* < 0.01; **D**, Incidence of childhood type 1 diabetes mellitus in three sections of sunshine durations: ^#^showed 3–4 hours/day vs. other two sections, both *p* < 0.01; all *p* derived from one-way ANOVA).

Overall incidence in different regions was indicated as following: Europe, 13.93 (12.59–15.27) per 100,000 children/yr; Asia, 4.31 (2.37–6.26) per 100,000 children/yr; North America, 21.75 (13.79–29.70) per 100,000 children/yr; South America, 4.47 (3.06–5.88) per 100,000 children/yr; Africa, 7.38 (4.37–10.39) per 100,000 children/yr; Central America and West Indies, 6.71 (3.27–10.16) per 100,000 children/yr; and Oceanic, 16.47 (13.67–19.27) per 100,000 children/yr; North America vs. other regions showed *p* < 0.01 excluded Oceania (Fig. 2A).

#### Incidence of CT1DM in different age-groups

Incidence of CT1DM in different age-groups as following: 0–4 years old, 9.70 (7.60–11.81) per 100,000 children/yr; 5–9 years old, 16.68 (12.51–20.86) per 100,000 children/yr; 10–14 years old, 20.27 (14.94–25.60) per 100,000 children/yr; 0–4 years old vs. 5–9 years old, *p* > 0.05; 5–9 years old vs. 10–14 years old, *p* > 0.05; 0–4 years old vs. 10–14 years old, *p* < 0.001 (Table 2, Fig. 2B).

#### Incidence of CT1DM in different climates type

Different gender for CT1DM incidence of different climates was displayed as follow: Monsoon climate: boy, 1.56 (0.95–2.16) per 100,000 children/yr; girl, 2.10 (1.28–2.92) per 100,000 children/yr; Oceanic climate: boy, 16.31 (14.29–18.33) per 100,000 children/yr; girl, 15.32 (13.51–17.12) per 100,000 children/yr; and the incidence of CT1DM of different genders in other climates showed in Table 3, all *p* > 0.05. Furthermore, overall incidence of different climates was presented as following: Mediterranean climate, 10.56 (5.69–12.42) per 100,000 children/yr; Monsoon climate, 2.12 (1.29–2.94) per 100,000 children/yr; Oceanic climate, 15.73 (13.93–17.54) per 100,000 children/yr; Continental climate, 12.30 (13.93–17.54) per 100,000 children/yr; Desert climate, 11.04 (7.06–15.02) per 100,000 children/yr; Savanna climate, 6.47 (2.68–10.26) per 100,000 children/yr; Rainforest climate, 7.58 (1.86–13.29) per 100,000 children/yr; pairwise comparison, Monsoon climate vs. other climates that excluding Savanna climate and Rainforest climate, all *p* < 0.01 (Fig. 2C).

**Table 3:** The incidence of childhood type 1 diabetes mellitus (per 100,000 children/yr) with different gender in different climate.

	Incidence			P*
	Boy	Girl	Total	
Mediterranean Climate	11.46 (9.11–13.81)	10.58 (8.81–12.35)	10.56 (8.69–12.42)	>0.05
Desert Climate	9.03 (5.76–12.29)	9.18 (5.39–12.98)	11.04 (7.06–15.02)	
Oceanic Climate	16.31 (14.29–18.33)	15.32 (13.51–17.12)	15.73 (13.93–17.54)	
Monsoon Climate	1.56 (0.95–2.16)	2.10 (1.28–2.92)	2.12 (1.29–2.94	
Continental Climate	12.34 (8.98–15.69)	12.75 (8.77–16.73)	12.30 (13.93–17.54)	
Savanna Climate	5.47 (1.01–9.93)	6.74 (1.96–11.52)	6.47 (2.68–10.26)	
Rainforest Climate	6.11 (0.88–11.35)	6.44 (0.30–12.58)	7.58 (1.86–13.29)	

Data showed as Mean (95% CI); *represented boy vs. girl, all *p* > 0.05, *p* derived from the *t*-test.

#### Incidence of CT1DM in countries with different sunshine durations

Incidence of CT1DM in countries with different sunshine durations as following: 3–4 hours/day, 15.17 (11.14–19.20) per 100,000 children/yr; 4–5 hours/day, 8.77 (5.71–11.84) per 100,000 children/yr; above 5 hours/day, 6.96 (4.07–9.85) per 100,000 children/yr; 3–4 hours/day vs. other sunshine durations, *p* < 0.01; 4–5 hours/day vs. above 5 hours/day, *p* > 0.05 (Fig. 2D).

#### Incidence of CT1DM in centers with different latitude

**Figure 3:**
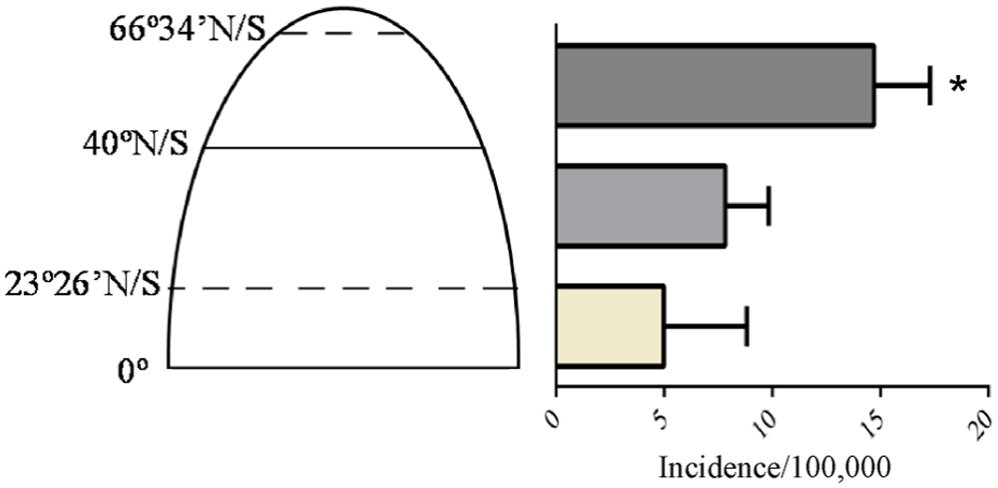
Incidence of childhood type 1 diabetes mellitus in three latitude sections (*expressed 40°–66°34′N/S vs. other two latitude sections, both *p* < 0.01, *p* derived from the one-way ANOVA).

Incidence of CT1DM in centers with different latitude as following: 0°–23°26′N/S: 4.98 (2.14–8.83) per 100,000 children/yr; 23°26′–40° N/S: 7.83 (6.01–9.84) per 100,000 children/yr; 40°–66°34′N/S: 14.71 (12.30–17.29) per 100,000 children/yr; 40°–66°34′N/S vs. other latitude, both *p* < 0.01; 0°–23°26′N/S vs. 23°26′–40° N/S, *p* > 0.05 (Fig. 3).

#### Incidence of CT1DM during different periods

**Figure 4:**
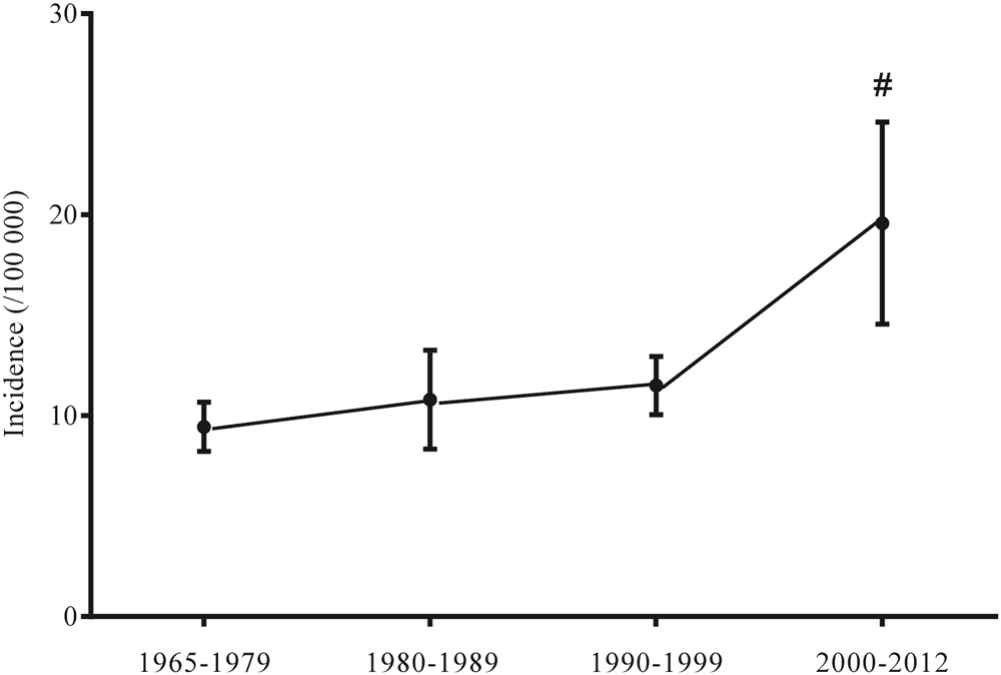
Incidence of childhood type 1 diabetes mellitus among four stages (*revealed 2000 to 2012 vs. other two stages, all *p* < 0.01, *p* derived from the one-way ANOVA).

Incidence of CT1DM during different periods as following: 1965 to 1979, 9.44 (8.22–10.66) per 100,000 children/yr; 1980 to 1989, 10.79 (8.33–13.26) per 100,000 children/yr; 1990 to 1999, 11.50 (10.04–12.95) per 100,000 children/yr; 2000 to 2012, 19.58 (14.55–24.60) per 100, 000 children/yr; 2000 to 2012 vs. other two groups, *p* < 0.01; 1965 to 1990 vs. 1990 to 1999, *p* > 0.05 (Fig. 4).

### Data availability

The datasets generated during and/or analyzed during the current study are available from the corresponding author on reasonable request.

## Discussion

This study performed firstly systematic estimates of CT1DM incidence among various climates, regions, genders, age-groups, latitude, and sunshine durations. The total countries based on this research consisted of 32% of the all countries in the world.

The worldwide incidence of CT1DM was increasing between 1965 and 2012 according to this study. Interestingly, the results of this study suggested there no significant difference was found in CT1DM incidence trended in boys compared with girls in this study. There are consistent results on the difference in incidence of diabetes by gender. Haynes *et al*. and Stipancic *et al*.^23,24^ displayed a significant increase incidence of T1DM in both boys and girls, and no significant difference was found in boys versus girls. However, others found a higher incidence in girls^25–27^. Adverse to our findings, Casu *et al*.^28^ expressed that a higher incidence of T1DM in boys. These divergences might depend on difference in sample size and statistical analysis.

In addition, our study illustrated a significantly increased incidence of T1DM in North America. Most countries in North America are developed countries with a higher per capita GDPs. Muntoni *et al*.^29^ showed that countries with a higher per capita GDPs tended to have higher T1DM incidence. Populations in wealthier countries typically drank more milk or eat more cheese than in poorer countries^30,31^. A high frequency of intake of milk or foods rich in protein may induce the occurrence and development of diabetes in humans^10–12,32^. Furthermore, these foods and dinks contain higher proportion of carbohydrate. Studies manifested that dietary carbohydrate could exacerbate postprandial glucose responses, which may be play a key role in blood glucose control^33,34^. Therefore, the higher proportion of carbohydrate may be also a major factor in development of diabetes in these regions.

Furthermore, the incidence of CT1DM of Asia has been increasing in recent years, although lower compared with Europe and America. Especially, the result of this study indicated the CT1DM incidence was higher in inland regions with continental climates compared with monsoon climates in China. The study stated the incidence of Huhehot is about 11 times in Hainan^4^. The higher incidence existed in inland region with high latitude that plays an important role in reducing childhood insulin-dependent diabetes mellitus (IDDM)^35^. Recent years, the per capita milk consumption and protein intake are increasing, especially in Xinjiang^36^ or Nnner Mongolia^37^. However, the overall incidence is low in China may based on individual’s diet habit and environmental factors^38^, which may resulted in a lack of public awareness, so could lead to a low quality of life of children in China.

As well, the incidence of CT1DM in regions with higher latitude and lower sunshine durations was higher than low latitude with high sunshine durations. In this study, the average incidence of CT1DM in Finland was 38.11 from 1965 to 1999, in which, latitude was 60°10′ N and the average amount of sunshine durations was only 3.18 hours a day. Eurodiab ACE Study Group^39^ had reported a 3-fold incidence increase of childhood IDDM was observed with the increasing latitude in Europe, and a similar result was reported within China^40^. In December, the northern Finland only has 2 hours of sunshine durations every day. Although there exists 23 hours of daylight per day in June, the most of the year exposure to daylight, Vitamin D production in the skin, is low by contrast with southern areas. Vitamin D supplementation is, thus, possibly more significant in this populations than others^41^. In this research, children lacked of adequate Vitamin D, who lived in higher latitude with low sunshine durations. Vitamin D is an immunosuppressive agent^42^, and the study believed the adequate Vitamin D supplementation for children might inhibit autoimmune reaction via damaging the β cells of pancreas and reduce the increasing trend in T1DM^41^. On the contrary, Vitamin D deficiency might induce CT1DM.

Last but not least, the incidence of CT1DM in centers or countries with oceanic climate was higher than other climates. The oceanic climate generally features long, but relatively mild winters and cool and short summers, which have a mean temperature below 22 °C in the warmest month^43^. In coastal areas of the higher middle latitudes (45–60° latitude), the prevailing onshore flow creates the basic structure of most oceanic climates. The previous studies reported the incidence rates of T1DM were associated with geographic variables such as average annual temperature^35^. Muntoni *et al*.^29^ indicated that countries or centers with lower annual temperatures tended to induce high incidence rate of CT1DM.

Nevertheless, this study just researched the incidence of CT1DM in 0–14 years old. Incidence data in older age groups exist from a few individuals. Furthermore, the incidence of childhood is unavailable after 2012 in this study. As well, the incidence of gender missing from Table 1 revealed the populations where development of the new register strategy was desired. Therefore, the continuous community-based registries are needed to access the T1DM incidence in the world, and further research is needed to find out the primary factor to identify prevention measures to stop the increased incidence of CT1DM.

## Conclusions

In this study, the worldwide incidence of CT1DM was increasing, especially in countries with oceanic climates. Compared with previous researches, other than milk consumption, per capita GDPs, and genders, we found the climates included latitude and sunshine durations might play a key role in inducing CT1DM, which affected the lifestyle and dietary habit of individuals.
